# Biological Approaches for Extraction of Bioactive Compounds From Agro-industrial By-products: A Review

**DOI:** 10.3389/fbioe.2021.802543

**Published:** 2022-01-27

**Authors:** Ailton Cesar Lemes, Mariana Buranelo Egea, Josemar Gonçalves de Oliveira Filho, Gabrielle Victoria Gautério, Bernardo Dias Ribeiro, Maria Alice Zarur Coelho

**Affiliations:** ^1^ Department of Biochemical Engineering, School of Chemistry, Federal University of Rio de Janeiro (UFRJ), Rio de Janeiro, Brazil; ^2^ Goiano Federal Institute, Rio Verde, Brazil; ^3^ School of Pharmaceutical Sciences, São Paulo State University (UNESP), Araraquara, Brazil

**Keywords:** enzyme extraction, fermentation, health benefits, bioactivities, agroindustrial

## Abstract

Bioactive compounds can provide health benefits beyond the nutritional value and are originally present or added to food matrices. However, because they are part of the food matrices, most bioactive compounds remain in agroindustrial by-products. Agro-industrial by-products are generated in large quantities throughout the food production chain and can—when not properly treated—affect the environment, the profit, and the proper and nutritional distribution of food to people. Thus, it is important to adopt processes that increase the use of these agroindustrial by-products, including biological approaches, which can enhance the extraction and obtention of bioactive compounds, which enables their application in food and pharmaceutical industries. Biological processes have several advantages compared to nonbiological processes, including the provision of extracts with high quality and bioactivity, as well as extracts that present low toxicity and environmental impact. Among biological approaches, extraction from enzymes and fermentation stand out as tools for obtaining bioactive compounds from various agro-industrial wastes. In this sense, this article provides an overview of the main bioactive components found in agroindustrial by-products and the biological strategies for their extraction. We also provide information to enhance the use of these bioactive compounds, especially for the food and pharmaceutical industries.

## 1 Introduction

The world produces large amounts of agroindustrial raw materials, mainly used for human and animal consumption and energy production (FAO, 2017; Sadh et al., 2018a). However, losses of up to 50% of the raw materials are estimated and occur mainly during harvest, post-harvest, slaughter, transport, processing, storage, and consumption (Arah et al., 2016; Lemes et al., 2020a). The losses can represent about 680 billion dollars per year (Dora et al., 2020) and correspond to about 25%–35% of the food produced in the world. These losses of raw material can be equivalent to 1.3 billion tons a year of material that is no longer consumed or transformed from appropriate processes (Ishangulyyev et al., 2019).

Due to its composition, residues can show slow degradability, resulting in accumulation and negative environmental impact (Sadh et al., 2018a). Thus, it is relevant to identify new applications to convert these residues into high-value-added products (Irmak, 2017). In general, agro-industrial residues present considerable concentrations of compounds such as fibers, lipids, carbohydrates, peptides, carotenoids, phenolic compounds, and other compounds, which have multiple functionalities and bioactivities and can be applied as ingredients in other products (Varzakas et al., 2016; Coman et al., 2020; Lopes and Ligabue-Braun, 2021).

The bioactive compounds present in the residue matrices can be potentially used in the prevention and treatment of several diseases, such as hypertension (Oliveira Filho et al., 2020), diabetes (Valencia-Mejía et al., 2019), cardiovascular disease (Rangel-Huerta et al., 2015), and neurological disease (Mohd Sairazi and Sirajudeen, 2020). In addition, bioactive compounds can be incorporated into foods, increasing their nutritional, sensorial, and technological properties (e.g., water and oil holding capacities, foaming, emulsion, and gelatinization) (Egea et al., 2018; Guimarães et al., 2019).

The proper use of agro-industrial matrices requires the production/extraction of bioactive compounds through ecofriendly strategies instead of conventional processes, followed by optimizing process conditions (Lemes et al., 2016a; Heemann et al., 2019). In this context, biological processes stand out, as they can enhance the production, extraction, and application of components from agro-industrial matrices in a more attractive way (Jegatheesan et al., 2020). Due to their selectivity, biological strategies present some advantages, including the production of extracts with high quality and bioactivity, as well as low toxicity (Chen, 2015; Habeebullah et al., 2020; Wang and Lü, 2021). Among the biological approaches, one consists of (1) extraction using enzymes that release compounds from the matrix under optimized conditions, making the process efficient (Marathe et al., 2019), and another is the (2) fermentation using different microorganisms that transform waste into products of interest, such as ethanol, proteins, peptides, enzymes, and pigments (Sadh et al., 2018b; Martínez-Espinosa, 2020).

In this sense, this article provides an overview of the main bioactive components found in agro-industrial by-products and the biological strategies for their extraction. We also provide information to enhance the use of these bioactive compounds, especially for the food and pharmaceutical industries.

## 2 Generation of Agro-industrial Waste

The agroindustry generates large amounts of waste regardless of the production chain step (Palhares et al., 2020; Chauhan et al., 2021). This waste generation can impact the environment according to factors that include the degree of development of the countries, education, population awareness, public policies, overexploitation, and waste of natural resources (Bedoić et al., 2019; Palhares et al., 2020). For example, in the steps involving food processing, losses reaching up to 40% of production are verified, mainly due to inefficiency in the production processing and management system, deformed or damaged products, and packaging disposable, among others, which generate refusal on the part of consumers (Dora et al., 2020).

**FIGURE 1:**
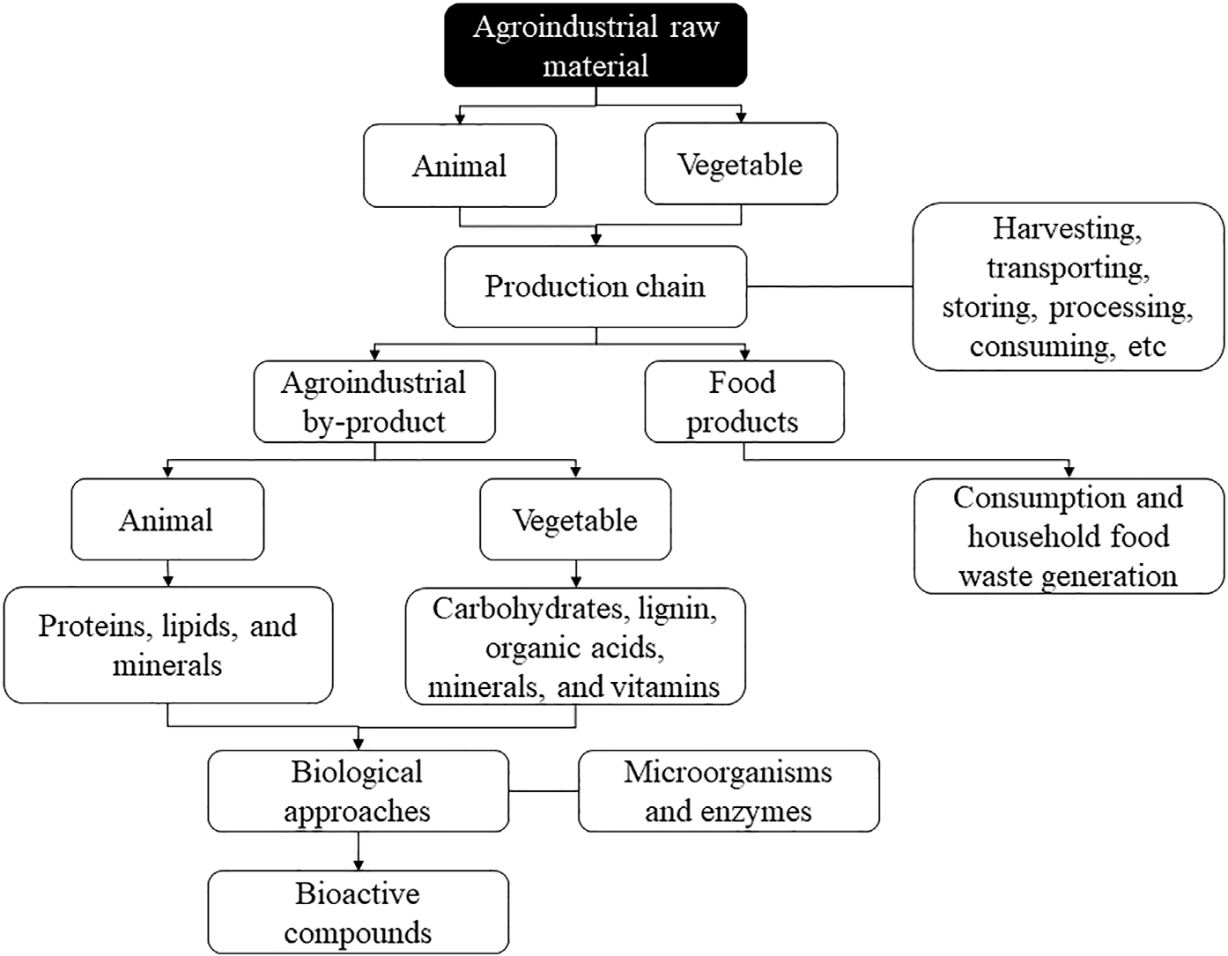
General steps that involve the generation of agroindustrial wastes and their use to produce bioactive components.

Waste can be from plant or animal sources (Figure 1). The vegetable by-products include leaves, stems, seeds, bark, straw, fibers, bagasse, and fruit skins, among others (Ezejiofor et al., 2014). For fruits and vegetables, for example, the production of industrial solid waste is verified, which includes items removed from fruits and vegetables during cleaning, processing, cooking, and packaging (EPA, 2012). For cereals, the waste generation corresponding to 35% of the total production is verified, including liquid residues (rice milling wastewater, parboiled rice effluent, corn steep liquor, and bakery wastewater) and solid wastes (corn pericarp, corn grits, and brewer’s spent grain), which are highly polluting due to large amounts of organic load, solid waste, and nutrients (Hassan et al., 2021). In general, vegetable residues present high carbohydrates (starch, cellulose, and hemicellulose), lignin, organic acids, minerals, and vitamins (Kumar et al., 2020).

On the other hand, animal by-products comprise large amounts of carcasses, skins, hooves, heads, feathers, viscera, bones, fat, meat trimmings, blood, and other animal fluids (Ockerman and Hansen, 2000; Waldron, 2007), as well as meat out of specification and significant amounts of milk processing residues such as whey and other fractions from the separation process (Ben-Othman et al., 2020). The meat sector, for example, records losses of up to 23% of everything produced, including consumption losses, industrial processing, distribution, inadequate storage conditions, and failures in the freezing process (Karwowska et al., 2021). The dairy industry generates around 4 to 11 million tons of waste per year, including whey, dairy sludge, and wastewater (processing, cleaning, and sanitary), with great pollutant potential (Ahmad et al., 2019; Lemes et al., 2020a). In general, animal residues present high levels of proteins, lipids, and minerals (Jayathilakan et al., 2012; Jain and Anal, 2016; Maysonnave et al., 2020).

Due to the complex chemical composition, animal and vegetable residues can be used as a low-cost raw material to obtain bioactive compounds using suitable processes (Lemes et al., 2016a; Prado et al., 2020).

## 3 Bioactive Compounds

Bioactive compounds can be used with functions like to (1) improve quality in conventional food (nutritional, sensory, and technological properties), (2) produce functional foods that provide physiological benefits in terms of essential nutritional aspects, (3) produce nutraceuticals, isolated components of food or agroindustrial wastes that provide proven physiological benefits (Birch and Bonwick, 2018; Daliu et al., 2018; Aguiar et al., 2019; Reque and Brandelli, 2021), (4) and compose films for application as smart, active, and/or bioactive food packaging (Nogueira et al., 2020; Oliveira Filho et al., 2021). This wide application of bioactive compounds occurs due to several effects attributed to bioactive compounds, including protection of the immune system, anti-inflammatory action, reduction of damage from cell oxidation, and the occurrence of chronic noncommunicable diseases (Silva et al., 2019; Alongi and Anese, 2021).

Several wastes can be used to obtain bioactive compounds, including cereal bran, which is rich in phenolic compounds, flavonoids, glucans, and pigments (Pauline et al., 2020); fruit and vegetable wastes, which are sources of phenolic compounds (Trombino et al., 2021); and complex carbohydrates (Pérez et al., 2002), as well as animal wastes, e.g., fish wastes rich in omega 3 (Bonilla-Méndez and Hoyos-Concha, 2018) and milk processing wastes as sources of peptides (Pires et al., 2021).

Among the main bioactive compounds found in agro-industrial wastes with more interest for application in the food and pharmaceutical industries are (1) bioactive peptides, (2) phenolic compounds, (3) carbohydrates, and (4) other molecules with distinct biological and technological properties. Below we describe these bioactive compounds of interest.

1) Bioactive peptides are protein fragments with up to 20 amino acid residues and that have an impact on body functions, which depend on their composition and amino acid sequence in the structure (Lemes et al., 2016b). Due to their high protein value, cakes and meals can serve as a source of peptides or amino acids that, once released, demonstrate higher biological activity such as antioxidant, antihypertensive, anti-inflammatory, and immune-modulating activities (Lemes et al., 2016b; Lemes et al., 2020b; Velliquette et al., 2020).

Peptides with antioxidant activity exert biological effects on the human body and have attracted great interest for their safety and wide distribution (Brandelli et al., 2015). When applied directly to food, peptides can decrease the occurrence and speed of oxidation reactions, which is especially interesting in replacing synthetic antioxidants related to toxic effects on human health. Currently, studies report the antioxidant activities of plant-derived hydrolysates, such as soybean (Yang et al., 2019), sunflower (Prado et al., 2020), corn (Zhang et al., 2019), beans (Paula et al., 2020), and peanut flour (Yu et al., 2021), as well as hydrolysates from animal by-products such as fish (Hemker et al., 2020) and poultry (Bouhamed et al., 2020).

The antihypertensive property has been mentioned for several peptide molecules with the potential to inhibit the activity of renin, angiotensin-converting enzyme, and angiotensin II receptors *in vitro* and *in vivo*, increasing the levels of nitric oxide in the blood (Lemes et al., 2020b). As a result, peptides show potential for application in antihypertensive prevention and treatment, reducing cardiovascular complications, mainly when associated with physical activity and a healthy diet.

2) Phenolic compounds are one of the main groups of secondary metabolites produced by plants and are of particular interest due to their bioactive properties such as antioxidant, antihypertensive, and antimicrobial activities and inhibition of carcinogenesis (Tanase et al., 2014; Tanase et al., 2019). In addition, phenolic compounds have been suggested for applications in food as active agents to control lipid oxidation and microbial growth in foods (Huang et al., 2020; Zhang et al., 2020) and in the pharmaceutical and cosmetic industries such as mouthwashes, eye creams, and different herbal cosmetics (Petti and Scully, 2009; Saraf and Kaur, 2010; Gaur and Agnihotri, 2014).

3) Carbohydrates are an important energy source and play numerous key roles in all living organisms (Jiang et al., 2021). They are at high levels in residues of vegetable origin, especially starch, lignocellulose (cellulose, hemicellulose, and lignin), and β-glucans, among others (Kumar et al., 2020). Starch is the main storage carbohydrate in plants and is a mixture of two glucose polymers: amylose and amylopectin (Lovegrove et al., 2017). Starches are used in various sectors of the industry in a wide range of products besides food applications (Di-Medeiros-Leal et al., 2021). Lignocellulose is the main polymeric compound formed by plant metabolism as structural material and is widely present in agro-industrial waste. This carbohydrate type has variable amounts of cellulose, hemicellulose, and lignin and can be converted into different high-value products, contributing to waste reduction (Fortunati et al., 2016). β-Glucan is a polysaccharide with several biological activities with scientifically proven beneficial health effects. Cereal wastes from barley, oats, and residual yeast biomass can also be used as a source of β-glucan (Tosh et al., 2010; Du et al., 2014; Vieira et al., 2017; Guedes et al., 2019; Liu et al., 2021).

4) Other molecules can be obtained, including lipid molecules such as lycopene-type carotenoids that can act as natural pigments in their application in foods and fatty acids.

Lycopene is a carotenoid, not a vitamin A precursor, mainly found in tomatoes and by-products of tomato processing (Anarjan and Jouyban, 2017). In addition to acting as a natural pigment giving a red-orange color, lycopene can react to free radicals, preventing cellular compounds’ degradation, including DNA (Moritz and Tramonte, 2006; Caseiro et al., 2020; Adetunji et al., 2021). All fractions of tomato can be used, including the skin, which contains about 510–734 mg of lycopene/kg of dry matter (DM), in addition to significant amounts of lutein, β-carotene, and *cis*-β-carotene (Knoblich et al., 2005; Nour et al., 2018), and the seed, which has a lycopene content of ∼130 µg lycopene/kg DM (Knoblich et al., 2005). Solid fractions can also be used for lycopene extraction (Trombino et al., 2021) from the use of solvents, supercritical extraction (Machmudah et al., 2012; Urbonaviciene and Viskelis, 2017; Hatami et al., 2019), pulsed electric field-assisted extraction (Pataro et al., 2020), and ohmic technology (Coelho et al., 2019), among others. In addition to lycopene, tomato by-products contain tocopherols, sterols and terpenes, fatty acids, phenolic compounds, and flavonoids, showing great versatility in obtaining several bioactive compounds (Kalogeropoulos et al., 2012).

Polyunsaturated fatty acids (PUFAs) of the omega-3 and omega-6 types are in vegetable oils, fish, and nuts. As with marine products, vegetable and nut by-products can be a source of PUFA that is underutilized. Fish by-products that are sources of marine oils can be used in the production of enzymatic PUFA synthesis of acylglycerols directly from glycerol and omega-3 fatty acid concentrates. The main acids present in omega-3 are docosahexaenoic acid (DHA), eicosapentaenoic acid (EPA), and α-linolenic acid, while acids present in omega-6 are arachidonic and linolenic acids (Dave and Routray, 2018).

## 4 Biological vs. Non-Biological Approaches

The recovery of compounds of interest from agricultural wastes and by-products involves conventional and novel solid–liquid and liquid–liquid extractions. Conventional methods are based on the extraction capacity of different solvents and applying thermal factors and/or homogenization, such as maceration, infusion, Soxhlet extraction, and hydro-distillation. However, these methods have presented some disadvantages such as long duration in the case of solid–liquid extraction, e.g., low extraction selectivity and specificity, decomposition of thermolabile compounds, and low purity of the product after the purification process, as well as high operation pressure, energy need, and amount of solvent with high purity (Gligor et al., 2019; Becerra et al., 2021).

On the other hand, novel extraction methods had been proposed, such as substituting molecular solvents with ionic liquids, eutectic solvents, and supercritical fluids or using different nonthermal energies (ultrasound, microwave, and pulsed electric field). However, the cell wall of plant matrices (and their components, cellulose, hemicellulose, starch, pectin, lignin, and proteins) can make the extraction of compounds a challenge.

In biological conversion processes (enzymatic or fermentation), cell wall recalcitrance is a resistance of plant cell walls to biological deconstruction for enzymes and microorganisms (Zeng et al., 2017) that varies among plant species and phenotypes (Silveira et al., 2013). However, extensive research has been carried out to establish effective protocols for pretreatment of cell wall material, such as lignocellulose, before using the waste for biological conversion (Baruah et al., 2018; Mankar et al., 2021). Available pretreatments include physical (milling, microwave, extrusion, and ultrasonication), chemical (alkali, acid, ionic liquids, organosolv, and deep eutectic solvents), physicochemical (steam, ammonia and CO_2_ explosion, and liquid hot water), and biological (whole-cell and enzymatic pretreatment) methods (Baruah et al., 2018).

Biological processes using microorganisms or enzymes can hydrolyze molecules, disrupt cell walls, increase permeability, and allow intracellular materials to be accessible for extraction. The microorganisms can utilize agricultural wastes as substrates under specific pH, temperature, moisture, and water activity conditions for their growth and production of the compounds of interest. The use of enzymes from microorganisms, plants, and mammalian cells and tissues—which catalyze reactions with high specificity, regioselectivity, and mild conditions—could improve the extraction efficiency of different compounds (polyphenols, carotenoids, terpenoids, and others) or even convert this compounds into valuable compounds as biofuels, surfactants, and pharmaceuticals (Gligor et al., 2019; Marathe et al., 2019; Becerra et al., 2021; Sharma et al., 2021).

### 4.1 Extraction of Bioactive Components With Enzymes From Agro-industrial Wastes

The enzyme-assisted extraction (EAE) method can be employed in pretreatments of raw materials, improving extraction time, solvent use, and the quality and purity of a product while lowering production costs compared with classical extraction processes. Different enzymes could be applied including cellulases, hemicellulases, pectinases, amylases, proteases, and lipases, as free or immobilized forms. The enzyme behavior depends on operational conditions such as pH, temperature, enzyme and substrate concentration, solid/liquid ratio, the particle size of the substrate, and reaction time (Becerra et al., 2021).

Among the advantages related to enzyme’s use at the industrial scale is the cost reduction since enzymes acting as catalysts provide process savings compared to conventional strategies (Singh et al., 2016). To further reduce the cost of applying processes using enzymes, agro-industrial waste available in large quantities can be used in the production of enzymes (Lemes et al., 2016a) using simpler purification protocols or coupling techniques to purify the target product (Lemes et al., 2014; Lemes et al., 2019). Another factor that supports the application of enzymes and cost reduction in the process is the possibility of their immobilization, resulting in their recyclable use, allowing their application in defined cycles, and maintaining their selectivity, catalytic activity, and the generation of products in large quantities (Braga et al., 2014; Basso and Serban, 2019).

Greener processes have also been proposed by combining enzymes with ultrasound, microwave, and alternative solvent-based extraction methods, which can result in higher product quality, decreased production costs and solvents, or increased enzymatic treatment efficiency and extraction yields. These complementary treatments may be employed before or after EAE and simultaneously with the process, and their features consist of shortened extraction periods, nontoxicity, non-flammability, use of recyclable solvents, overall simplified steps, and customizable process parameters (Gligor et al., 2019; Wen et al., 2020; Picot-Allain et al., 2021). Table 1 shows examples of the use of enzymes in the extraction and recovery of these bioactive compounds from agro-industrial wastes.

**TABLE 1:** Examples of enzyme-assisted extraction of bioactive compounds from agroindustrial by-products.

Enzymes	Matrix/Metabolite	Extraction conditions	References
Pectinase, alpha-amylase, hemicellulase, cellulase, and glucoamylase	Guarana (*Paullinia cupana*) seeds/Caffeine and tannins	Solvent-biomass ratio: 5 ml/g, solvent: water, 50–70°C, enzyme loading: 0.1–1% v/v biomass, 5.5 h, 200 rpm	Ribeiro et al. (2012)
Proteases, pectinase, cellulase, and hemicellulase	Flaxseed meal/polyphenols and proteins	Solvent-biomass ratio: 6.58 ml/g, solvent: water or 10% ethanol v/v, 50°C, enzyme loading: 0.3–2.0%, v/v, 1.5 h, 200 rpm	Ribeiro et al. (2013)
Cellulase, glucosidase, and pectinase	Grape skins/Anthocyanins and flavanols	Solvent-biomass ratio: 20 ml/g, solvent:12.5% ethanol solution with 4 g/L tartaric acid, pH 3.6, 20–30°C, enzyme loading: 15 mg/L, 72 h	Nogales-Bueno et al. (2020)
Pectinase, and *α*- and *β*-Glycosidase	Grape pomace/aroma compounds (alcohols, esters, terpenes, and others)	Particle diameter: < 500 μm, Solvent-biomass ratio: 0.6 ml/g, solvent: 70% ethanol/Milli-Q water solution, pH 5.0, 35°C enzyme loading: 0.9 g/10 ml, 48 h, 120 rpm	Liang et al. (2020)
Protease	Blue crab (*Portunus segnis*) shells/carotenoproteins	Solvent-biomass ratio: 5 ml/g, solvent: water (pH 8.0), 50°C, enzyme loading: 20 U/g biomass, 60 min	Hamdi et al. (2020)
Proteases and cellulase	*Salvia officinalis* leaves/Rosmarinic acid	Solvent-biomass ratio: 25.76 ml/g, solvent: water (pH 6.9), 54.3°C, enzyme loading: 4.49%, w/w, 2 h with stirring	Su et al. (2020)
Pectinase	Spent coffee ground/flavonoids	Solvent-biomass ratio: 15 ml/g, solvent: sodium acetate buffer (200 mM, pH 5.5), 37°C, enzyme loading: 0.67% v/v, 60 min	Khairil Anuar et al. (2020)
Cellulase and hemicellulase	Japanese Peppermint (*Mentha arvensis*) leaves/essential oil	Solvent-biomass ratio: 10 ml/g, solvent: water, 40°C enzyme loading: 2%w/v, 3 h, 120 strokes/min	Shimotori et al. (2020)
Polygalacturonase, pectin lyase, celulase, and xylanase	Unsold ripened tomatoes/carotenoids	solvent: acetate buffer (100 mM, pH 5.5), 50°C, enzyme loading: 25 U/g, 180 min	Lombardelli et al. (2020)
Lysozyme	Spirulin (*Arthrospira platensis*)/C-phycocyanin	Solvent-biomass ratio: 8 ml/g, solvent: phosphate buffer (100 mM, pH 6.8), 37°C, enzyme loading: 0.6% w/v, 16 h + US: 20kHz, 50% amplitude, 2.5 min	Tavanandi and Raghavarao, (2020)
Proteases, hemicellulase, pectinase, and cellulase	Tiger nut (*Cyperus esculentus*)/oil	Particle diameter: < 600 μm, Solvent-biomass ratio: 10 ml/g, solvent: water, pH 4.9, 45°C enzyme loading: 2% w/v, 180 min, 120 rpm; MW: 2.45GHz, 300 W, US: 25 KHz, 460 W, 30 min, 40°C	Hu et al. (2020)
Polygalacturonase, celulase, and hemicellulases	*Opuntia ficus-indica* cladodes/isorhamnetin conjugates	Solvent-biomass ratio: 5 ml/g, solvent: ethanol/water 90/10, pH 4.0, 40°C enzyme loading: 1.5% w/v, 30 min, scCO_2_: pressure 100 bar, flow rate: 18 g/min, 10–40 min, 60°C, co-solvent: 20% ethanol	Antunes-Ricardo et al. (2020)
β-glucosidase, tannase, and cellulase	Citrus pectin by-product/aglycone flavanones	Solvent-biomass ratio: 12.5 ml/g, solvent: acetate buffer (20 mM, pH 5.0), 40°C, enzyme loading: 20 U/g biomass, 24 h, 120 rpm	Barbosa et al. (2021)
Cellulase, xylanase, and pectinase	Red beets/betalains	Solvent-biomass ratio: 15 ml/g, solvent: acetate buffer (pH 5.5), 25°C, enzyme loading: 24 U/g, 4 h	Lombardelli et al. (2021)
Cellulase, hemicellulase, and pectinase	Licorice roots/glycyrrhizic acid	Particle diameter: < 2 μm, Solvent-biomass ratio: 5 ml/g, solvent: acetate buffer, pH 5.0, 45°C enzyme loading: 2% w/v, 1 h with stirring	Giahi et al. (2021)
Beta-glucanase, pectinase, protease, and ferulic acid esterase	Sweet cherry (*Prunus avium*) pomace/polyphenols	Solvent-biomass ratio: 2.63 ml/g, solvent: sodium phosphate buffer (100 mM), pH 10.0, 70°C enzyme loading: 2–140 μl/g, 18.4–40 min, 750 rpm	Domínguez-Rodríguez et al. (2021)
Cellulase	Passion fruit/polyphenols	Particle diameter: < 180 μm, Solvent-biomass ratio: 50 ml/g, solvent: water, pH 5.0, 30°C enzyme loading: 6% w/v, 47 min, US: 50 kHz, 300 W	Wang et al. (2021)
α-Amylase, β-glucanase, protease, hemicellulases, lipase, phytase, cellulases, and pectinase	Mango peel/phenolic acids	Solvent: sodium phosphate buffer, pH 4.5–7.5, 37–63°C enzyme loading: 2.3–4.1% w/v, 60–120 min, US: 40 kHz, 45–120 W	Sharif et al. (2021)
Pectinases	Pomelo (*Citrus maxima*) peel by-products/flavonoids	Particle diameter: < 149 μm, Solvent-biomass ratio: 142.99 ml/g, solvent: water, enzyme loading: 3.45% w/v, 65.23 min + US: 40 kHz, 69.26 min, 30°C	Anh et al. (2021)
Cellulase, pectinase, and tannase	Olive pomace/polyphenols	Solvent-biomass ratio: 15 ml/g, solvent: water, pH 5.0, 60°C enzyme loading: 2% w/v, 17 min, 120 rpm, MW: 2.45 GHz, 600 W	Macedo et al. (2021)

### 4.2 Fermentation Processes as a Tool to Obtain Bioactive Compounds From Agro-industrial Wastes

**FIGURE 2:**
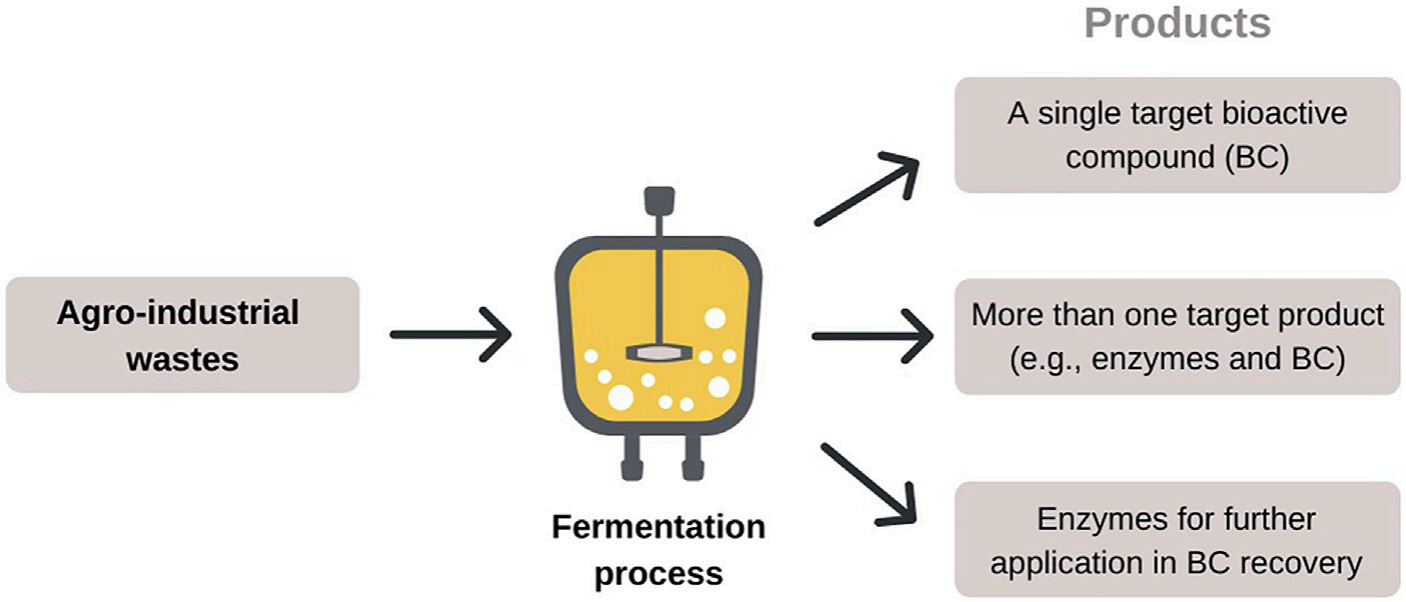
Perspectives about the fermentation process to obtain bioactive compounds (BC).

The fermentation process as a tool for obtaining bioactive compounds from agroindustrial wastes can be seen under different perspectives (Figure 2) as follows: (1) the target compound is the main product of microbial fermentation of agro-wastes, (2) the target compound is one of the products resulting from microbial fermentation of agro-wastes, and (3) microbial fermentation of agro-wastes produces enzymes, which will be applied to recover the target compound from a particular substrate.

#### 4.2.1 Production of Bioactive Compounds Using Fermentation of Agro-industrial Wastes

The production of bioactive compounds through fermentation can be carried out with various microorganisms and their respective species (bacteria, yeasts, filamentous fungi, and others) (Moreira et al., 2018; Shin et al., 2019; Gulsunoglu et al., 2020; Jiang et al., 2020; Silva et al., 2020; Sinha et al., 2021). In addition, wild or genetically modified microorganisms (Cipolatti et al., 2019; Yang et al., 2020) can be applied in fermentative processes to obtain high-added-value compounds. The fermentation product can be part of the cell metabolism of microbial species or be extracted from the substrate by the microorganism’s action. The target compound can also be produced by the microorganism intracellularly (Rodrigues et al., 2019)—which needs cell rupture steps after fermentation for the compound recovery (Gomes et al., 2020)—or extracellularly (Acosta et al., 2020).

Fermentative strategies differ between solid (SSF) and submerged (SmF) states. The choice of the fermentation process will depend on the used microorganism and the process recovery of target compounds. In the SmF approach, microorganisms are grown in a liquid medium containing the nutrients (Dey et al., 2016). The target compounds are secreted into the fermentation medium and then recovered in a separation step, such as centrifugation. SmF offers better control of cultivation conditions and is most suitable for bacteria and yeasts requiring high moisture content. SmF also allows the proper mixing of nutrients due to the high amount of free water and is a method of easy handling and scaling up. Nevertheless, the target products tend to be diluted at the end of fermentation (Bagewadi et al., 2018; Sánchez et al., 2021).

In contrast, SSF utilizes solid substrates in the absence or near absence of free water, which is a more appropriate condition for the growth of filamentous fungi (Soccol et al., 2017). Microbial growth and product formation occur on the surface of a solid substrate that works as support or on an inert material impregnated with nutrient solution (Thomas et al., 2013). After the SSF process, the target compounds are recovered through extraction and separation steps. SSF presents minimal problems with microbial contamination due to the low water contents in the medium and offers high volumetric productivity, concentrated target compounds, tolerance of high substrate concentration, and less wastewater generation (Manan and Webb, 2017; Krishania et al., 2018).

In both fermentation strategies, agro-industrial wastes can be utilized as a nutritional source for microorganism species to obtain bioactive compounds (Kaur et al., 2019; Reque et al., 2019; Abdeshahian et al., 2020; Jiang et al., 2020; Sharma and Ghoshal, 2020; Sinha et al., 2021). Regarding the complex matrices of some wastes, pretreatments are applied before the fermentation to facilitate the microorganism’s access to nutrients (Acosta et al., 2020).

The choice of substrate will depend on the nutritional needs of the microbial species to produce the target compounds. A combination of wastes from different sources can also be an alternative to supply the nutritional requirements for microbial growth (Otero et al., 2019). In addition, the fermentation of wastes to produce bioactive compounds can be optimized through some approaches including response surface methodology (Moayedi et al., 2018; Rodrigues et al., 2019; Abdeshahian et al., 2020; Yang et al., 2020) and one factor at a time (Amorim et al., 2019; Kaur et al., 2019). The main bioactive compounds that can be produced using the biological approach were highlighted, as well as the particularities of each process.

Protein hydrolysates with biological actives can be obtained using one-step fermentation from wastes (Mechmeche et al., 2017; Fontoura et al., 2019). Moayedi et al. (2016) established a fermentative process with the *Bacillus subtilis* strain to convert tomato waste proteins into antioxidant and antibacterial hydrolysates. Mechmeche et al. (2017) investigated the bioconversion of tomato seed meal extract into antioxidant peptides through a fermentative process with *Lactobacillus planetarium*, which showed a promising ability to degrade and convert tomato seed proteins into peptides, also contributing to the antioxidant activity of the hydrolysates. Maciel et al. (2017) explored the keratinolytic potential of *Chryseobacterium* sp. and *Bacillus* sp. to convert feathers into protein hydrolysates with better *in vitro* nutritional features, suggesting a good prospect for their use in animal feed. Similarly, Fontoura et al. (2019) obtained protein hydrolysates with antioxidant properties through SmF of feathers with *Chryseobacterium* sp. Jiang et al. (2020) proposed an optimized production of bioactive peptides with antioxidant activity by *B. subtilis* from corn gluten meals.

Some studies have investigated the production of phenolic compounds through bioconversion of wastes. Shin et al. (2019) evaluated the fermentation of black rice by *Aspergillus* species under SSF to produce antioxidant phenolic compounds. After 3 days of fermentation, a maximum production of 1,660 µg protocatechuic acid/g of substrate was achieved. The authors also pointed the requirement to pretreat the waste for the extraction of phenolic compounds. Reque et al. (2017) addressed the bioprocessing of wheat middlings by *Bacillus* sp. to increase its antioxidant phenolic compound content. Besides changes in the phenolic profiles, the bioprocessed wheat middlings exhibited higher antioxidant capacity and total phenolic amounts than the unfermented waste. In the same way, Gulsunoglu et al. (2020) evaluated the effect of SSF with four *Aspergillus* spp. as a strategy to enhance the contents of phenolic compounds of apple peels. As a result, the 7-day fermentation enhanced apple peels’ phenolic contents and antioxidant activity by between threefold and fivefold. The enhancement of antioxidant phenolic compounds through bioprocessing of by-products from fig (Buenrostro-Figueroa et al., 2017) and apricot (Dulf et al., 2017) is also reported. The bioprocessing of brewer’s spent grain can favor both its phenolic compound and bioactive peptides contents, thus contributing to its antioxidant activity (Verni et al., 2020).

Potential prebiotic oligomers can be produced under microbial fermentation of wastes (Amorim et al., 2019; Reque et al., 2019). Wheat middlings, a by-product from wheat flour production, showed good aspects to be utilized as a substrate for xylooligosaccharide (XOS) production by *B. subtilis,* demonstrating prebiotic activity through *in vitro* tests with *Lactobacillus acidophilus*, a commercial probiotic strain (Reque et al., 2019). Likewise, brewers’ spent grain was fermented by a genetically modified *B. subtilis* to produce arabino-xylooligosaccharides (AXOS), and after optimizing the fermentation process, AXOS with a degree of polymerization (DP) of 2–6 were obtained. Furthermore, AXOS yield using a genetically modified strain increased 33% in comparison to the wild type (Amorim et al., 2018). In both cases, the bioconversion of wastes into xylooligomers occurred due to the ability of strains to secrete xylanases, the main enzymes involved in XOS production. Yang et al. (2020) proposed an efficient preparation of oligogalacturonides (OGS) using fermentation of citrus peel wastes with an engineered *Pichia pastoris* strain. The one-step fermentation of mandarin and orange peel wastes resulted in OGS with a DP of 2–7 and 2–6, respectively, and a maximal OGS yield of 26.1% after process optimization.

β-Glucan, a polysaccharide with several biological activities, is commonly extracted from cereal by-products (Karimi et al., 2019) and brewing/winery spent yeasts (Pinto et al., 2015; Varelas, 2016). However, β-glucan can also be produced by fermentation processes from wastes. Abdeshahian et al. (2020) investigated the production of extracellular β-glucan from sugarcane straw by *Lasiodiplodia theobromae*. The highest β-glucan yield and productivity were 0.047 g/g glucose and 0.014 g/L·h, respectively, at 72 h of fermentation. Acosta et al. (2020) evaluated the use of soybean molasses (unhydrolyzed and hydrolyzed forms) as a raw material for the fermentative production of β-glucan by *L. theobromae*. Maximum β-glucan production (1.06 g/L) and yield (0.13 g/g) were obtained in fermentations using unhydrolyzed molasses. Bzducha-Wróbel et al. (2020) proposed the valorization of waste potato juice water for β-glucan preparation with the *Candida utilis* strain, resulting in a β-glucan yield of 63 g/100 g yeast dry weight after 72 h of fermentation.

Natural pigments as carotenoids can be produced through biotechnological processes from wastes (Cipolatti et al., 2019; Otero et al., 2019). Corn steep liquor and sugarcane molasses were used as substrates to produce carotenoids by the *Rhodotorula mucilaginosa* strain through batch and fed-batch fermentation. Among the two fermentation approaches evaluated, the fed-batch process increased the carotenoid production by 400% compared to the batch process (Rodrigues et al., 2019). Onion peels, potato skin, mung bean husk, and pea pods were also evaluated as substrates to produce carotenoids under SmF with *R. mucilaginosa*. These wastes were chosen based on the high concentrations of sugars (onion peels and potato skin) and nitrogen (mung bean and pea pods). As a result, it was found that onion peels and mung bean husk are potential substrates for the production of microbial carotenoids as β-carotene, phytoene, torulene, and torularhodin (Sharma and Ghoshal, 2020). Olive mill wastes (Ghilardi et al., 2020), coffee pulp and husk (Moreira et al., 2018), and wastes from the vegetable and fruit markets (Sinha et al., 2021) were explored in carotenoid production by *Rhodotorula* species, showing good prospects as cheap substrates. Orange, carrot, and papaya peels were used as substrates to produce β-carotene under SSF with *Blakeslea trispora*, resulting in good yields in synthetic media (Kaur et al., 2019).

#### 4.2.2 Low-Cost Microbial Enzymes for Further Recovery of Bioactive Compounds

Microbial enzymes produced by the fermentation of wastes are extensively applied to recover bioactive compounds (Zanutto-Elgui et al., 2019; Gautério et al., 2021a). Several studies have already demonstrated the effective use of wastes to obtain microbial hydrolases such as proteases, lipases, and carbohydrases (Pereira et al., 2019; Ahmad et al., 2020; Gautério et al., 2020).

The use of agro-industrial wastes reduces the production costs of microbial enzymes, being an alternative to replace synthetic and commercial substrates. The expenses related to the recovery of target compounds are also positively affected, as enzymatic extracts have a lower cost than pure commercial enzymes. Nevertheless, it is necessary to verify if the application requires purified enzymatic extracts since the inclusion of purification steps would result in a more costly process (Lemes et al., 2014; Lemes et al., 2019).

Zanutto-Elgui et al. (2019) produced bioactive peptides from bovine and goat milk subjected to the proteolytic activity of *Aspergillus oryzae* and *Aspergillus flavipes* proteases. Proteolytic enzymes from fungal species were effectively produced under SSF in wheat bran and then applied in the hydrolysis of milk proteins. The milk peptides showed broad antimicrobial and antioxidant activities *in vitro*, thus demonstrating a good prospect for biotechnological applications of these bioactive compounds. Oliveira et al. (2015) evaluated the production of soy protein hydrolysates with a microbial protease preparation. First, the protease production occurred using SmF from feather meal broth with the *Chryseobacterium* sp. strain. Then the enzymatic hydrolysis of soy protein isolate (SPI) occurred using the microbial protease extract, thus evaluating soluble peptides, antioxidant activity, and emulsifying capabilities of the hydrolysates. The authors pointed out that enzymatic hydrolysis increased soluble peptide content and positively affected SPI’s antioxidant and emulsifying properties. Moreover, the enzymatic treatment was demonstrated as a promising approach to obtain antioxidant compounds for food application, besides providing functional properties.

Gautério et al. (2020) utilized rice bran as a xylan source to produce xylanases by the *Aureobasidium pullulans* strain. In a subsequent study, crude and partially purified xylanases from *A. pullulans* CCT 1261 were applied in beechwood xylan hydrolysis, resulting in XOS, and the pretreatment did not influence the total concentration of XOS (Gautério et al., 2021a). The optimization of the hydrolytic process also demonstrated the successful use of crude xylanase to obtain XOS with low xylose release (Gautério et al., 2021b).

Kupski et al. (2018) applied a fungal cellulolytic complex to provide functional compounds—mainly proteins and phenolic compounds—from soybean meal (SBM) and corn husk (CH). The cellulolytic enzymes were obtained using SSF of rice husk and bran with *Rhizopus oryzae* CCT 7560 and then used to hydrolyze SBM and CH. Enzymatic hydrolysis resulted in 34% cellulose reduction in SBM, whereas, in CH, it was 55%. This reduction increased the protein (74%) and starch (95%) digestibility in SBM. In CH, in turn, the reduction allowed the release of phenolic compounds (21%). As mentioned by the authors, available protein in SBM can be used as a food supplement, whereas the phenolic contents from CH can be applied as a food additive.

#### 4.2.3 Simultaneous Production of Bioactive Compounds Using Fermentation of Agro-industrial Wastes

The use of agro-industrial wastes to produce bioactive compounds via fermentation strategies has undergone several advances. One of them refers to co-production, which is cost-efficient and meets the sustainable context of the circular economy. Some examples include the simultaneous production of antioxidant compounds and proteolytic enzymes (Lemes et al., 2016a); lignocellulolytic enzymes and phenolic compounds (Leite et al., 2019); xylanases and xylooligomers (Menezes et al., 2017; Pereira et al., 2018); proteolytic enzymes and protein hydrolysates (Bernardo et al., 2019); lipids and carotenoids (Kot et al., 2019; Costa et al., 2020; Silva et al., 2020); and antioxidant peptides and pigments (Bertolini et al., 2021), among others.

The co-production has some challenges to be overcome such as (1) optimization of the fermentation process, which becomes more complicated when the aim is to obtain the maximum yield of all the target compounds; (2) application of treatments to wastes, which can sometimes favor the production of only the target compound; and (3) separation of compounds produced, where questions related to the application and purity of the target compounds—use of a mixture or use of each compound separately—must be analyzed.

## 5 Downstream Processing of Bioactive Compounds From Agro-industrial Wastes

The recovery and purification of bioactive compounds are strongly related to the particularities of the target biomolecule and its future application. Some bioactive compounds’ characteristics, such as the nature of the compound, cell location (intracellular or extracellular), size, structure, charge, and solubility, among others, will determine the steps to be applied for its recovery and purification. Furthermore, the purity required of the target compound as well as the preservation of their bioactivity must also be considered when establishing the purification steps.

Using agro-industrial wastes to obtain bioactive compounds using biotechnological approaches also influences the subsequent recovery and purification steps. Waste features such as particle size, solubility, viscosity, and recalcitrance can interfere with the extraction, cell disruption, and purification of the target compound, in addition to determining the number of downstream steps. Another critical point is related to the separation of bioactive compounds produced simultaneously in the same medium. All these aspects determine the complexity and costs of purification designs (Lemes et al., 2014; Lemes et al., 2020a).

Bioactive compounds produced by enzymatic hydrolysis or SmF procedures can be separated from the medium using solid–liquid techniques (e.g., filtration and centrifugation). Target compounds produced by SSF need to be recovered by extraction. The extraction approaches applied to bioactive compounds include solvent extraction (e.g., organic, eutectic, and ionic liquid solvents), ultrasound-assisted extraction, microwave-assisted extraction, enzymatic-assisted extraction, pulsed electric field, subcritical and supercritical fluid extraction, aqueous two-phase system, and three-phase partitioning (Zainal-Abidin et al., 2017; Sagar et al., 2018; Yan et al., 2018). Some examples include the extraction of β-carotene (Kaur et al., 2019) and phenolic compounds (Gulsunoglu et al., 2020) using organic solvents after SSF of fruit and vegetable peels; the extraction of lycopene using organic solvent (ethanol) after enzymatic-assisted treatment of tomato by-products (Azabou et al., 2016); the extraction of phenolic compounds using ionic liquids (Magro and Castro, 2020) and ultrasound-assisted procedure (Ajila et al., 2011) after SSF of lentil grains and apple pomace, respectively; and the extraction of phenolic compounds from fermented orange pomace using supercritical CO_2_ and cosolvents (Espinosa-Pardo et al., 2017).

Regarding intracellular target compounds, a step of cell disruption or cell permeabilization and subsequent extraction procedure is required, and both can occur separately or simultaneously (Kalil et al., 2017). The cell disruption/destabilization techniques applied to releasing compounds include bead milling, ultrasonication, high-pressure homogenization, osmotic shock, freeze–thawing, and lysis procedures with enzymes, chemicals, and heating (Gomes et al., 2020).

Purification processes applied to bioactive compounds are diverse and range from low- to high-resolution techniques. Some of the mentioned procedures in the literature are aqueous-phase separation (Wang et al., 2019), membrane technology (Pezeshk et al., 2019; Singh et al., 2020), and chromatography (Moayedi et al., 2018; Fontoura et al., 2019). Each technique can be applied alone or combined in purification designs, considering the aspects of the target compounds mentioned above. The challenge is to establish a purification process that results in desirable compound purity in the highest yield possible (Lemes et al., 2014).

## 6 Potential Applications of Bioactive Compounds Recovered From Waste and By-products

The growing demand for foods with beneficial effects on health, while contributing to the sustainable use of natural resources, stimulates the use of by-products to obtain bioactive compounds (Vilas-Boas et al., 2021), which have multiple applications in food, acting as antimicrobials, antioxidants, natural dyes, fortifying ingredients, texture modifiers, and others (Veneziani et al., 2017).

**FIGURE 3:**
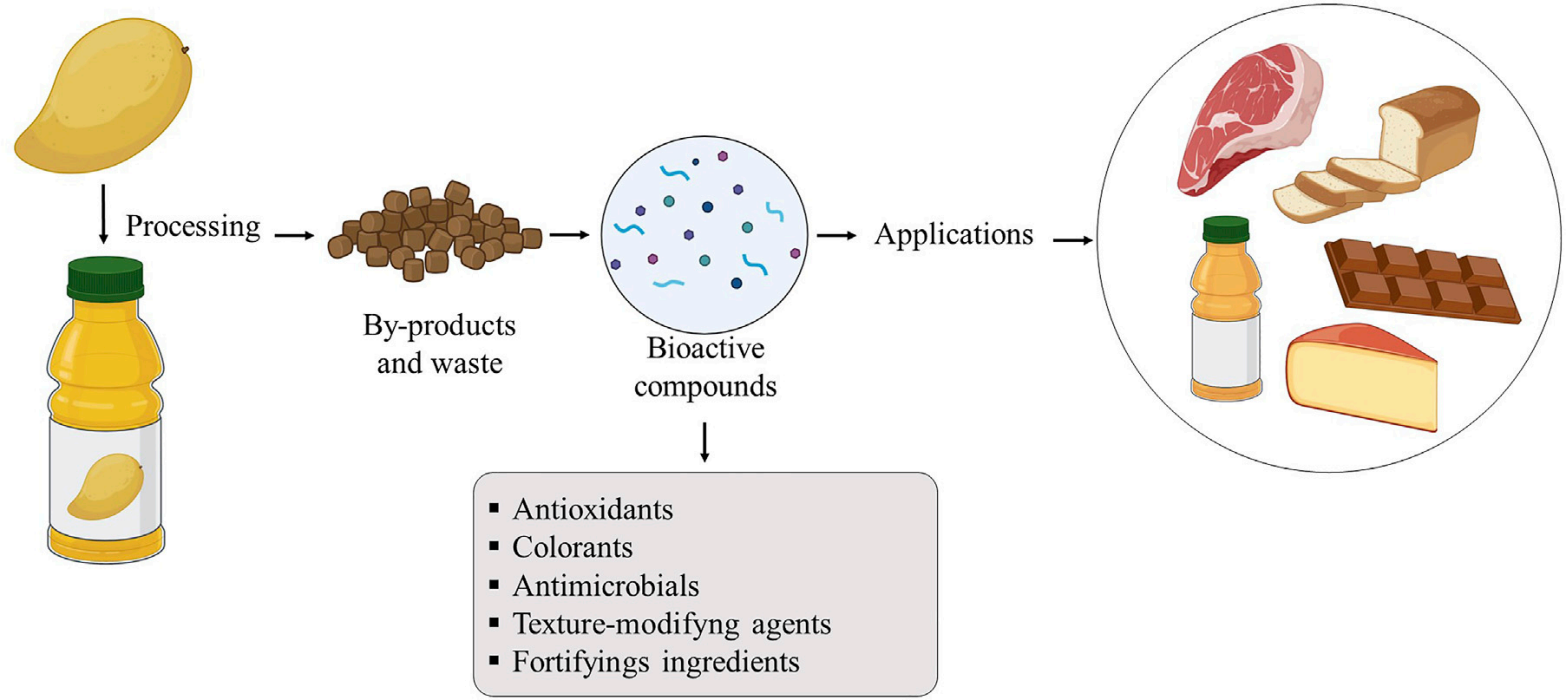
Potential applications of bioactive compounds recovered from by-products.

Figure 3 shows an overview of the potential application of bioactive compounds recovered from food by-products in meat, dairy, bakery, chocolate, and juice products, where the compounds are used as food ingredients with a defined role in the protection and technological properties of food.

A large number of studies report the addition of bioactive compounds from by-products in various food systems (Table 2), being applied as antioxidant and antimicrobial agents and also as agents to improve the nutritional and functional value of food products such as frozen fish (Özen et al., 2011), yogurt (Fidelis et al., 2020), dry cured sausage “chorizo” (Lorenzo et al., 2013), beef patties (Zamuz et al., 2018), bread (Rizzello et al., 2009; Peng et al., 2010), and petit Suisse cheese (Deolindo et al., 2019).

**TABLE 2:** Application of bioactive compounds in food products.

Bioactive compound	Addition levels	Food product	Formulation properties	References
Amaranthus spp. Seeds extract rich in antifungal peptides	7.04 and 22.96%	Bread	↑ nutritional value (protein and free amino acids)	Rizzello et al. (2009)
			Delay in the appearance of fungal mycelium in storage	
			No changes in taste and flavor	
Phenolics and carbohydrate fractions of okra seed and seedless pod	300 mg, 600 mg, and 1 g/500 g	Bread	↑ bread antioxidant activity	Peng et al. (2010)
			↓ antioxidant activity (30–40%) with thermal processing; ↓formation of harmful compound *N* ^*ε*^-(carboxymethyl)lysine	
			Acceptable color change acceptable with little effect on quality	
Grape seed extract powder	20 g CE/kg	Frozen fish	Inhibition of the formation of lipid hydroperoxides and thiobarbituric acid reactive substances (TBARS)	Özen et al. (2011)
Grape seed extracts	1 g/kg	Dry cured sausage “chorizo”	↓ oxidation determined using TBARS method and the total volatile compounds of lipid oxidation	Lorenzo et al. (2013)
			↑ sensory acceptance compared to those formulated with BHT, chestnut extract and control	
Hull, bur, and leaf chestnut extracts	250–1,000 mg/kg	Beef patties	↓ lipid oxidation in hamburgers	Zamuz et al. (2018)
			↑ reduction of metmyoglobin at higher doses	
			It did not affect sensory acceptance	
Grape seed extract	0.5 g/100 g	Petit Suisse cheese	↑ total phenolics and antioxidant activity (up to 28 days)	Deolindo et al. (2019)
			73% sensory acceptance	
			77% inhibition of angiotensin-converting enzyme (ACE) activity	
Camu-camu (*Myrciaria dubia*) seed extract	1.0 g/100 g	Yogurt	↑ antioxidant and chemical reducing capacity (FRAP, DPPH, and FCRC methods)	Fidelis et al. (2020)
			The camu-camu yogurt containing 0.25 g/100 g of lyophilized camu-camu (*Myrciaria dubia*) seed extract had an acceptance rate of 84%	

The antioxidant potential of extracts from by-products is frequently used in foods and has been mainly associated with their content of total phenolic compounds determined through different methods (Lorenzo et al., 2013; Zamuz et al., 2018; Fidelis et al., 2020) since the compounds have chemical properties and structural diversity that influences the mechanism of action associated with this bioactivity (Vuolo et al., 2019).

Phenolic compounds such as aliphatic alcohols, terpenes, acids, aldehydes, ketones, anthocyanins, and isoflavonoids are the main bioactive compounds in by-products with antimicrobial properties (Arshad and Batool, 2017) and, therefore, with potential for incorporation into food matrices. In addition to these compounds, water-soluble extracts rich in antimicrobial peptides can be obtained from agro-industrial by-products such as amaranth seed extract to inhibit fungal species isolated from bakery products, in which when applied to the bread matrix (gluten free and with bread flour wheat), the inhibitory activity is verified during the entire shelf life of the products (Rizzello et al., 2009).

In addition to the antioxidant and antimicrobial potentials, bioactive compounds may have other biological properties such as antiproliferative, antidiabetic, and antihypertensive activities (Ben-Othman et al., 2020). For example, Fidelis et al. (2020) observed that camu-camu seed extract has a high content of total phenolics (46.3% w/w), contain mainly vescalagin, castalagin, gallic acid, procyanidin A2, and (−)-epicatechin. This extract demonstrated high antioxidant and antiproliferative activities against HepG2 cells and Caco-2 cells, inhibiting α-amylase (40.7%), α-glucosidase (16.6%), and angiotensin-converting enzymes (34.4%). The application of the extract in yogurts increased the antioxidant capacity without affecting sensory acceptance (84%), an important factor for the application of any new ingredient in formulations.

**FIGURE 4:**
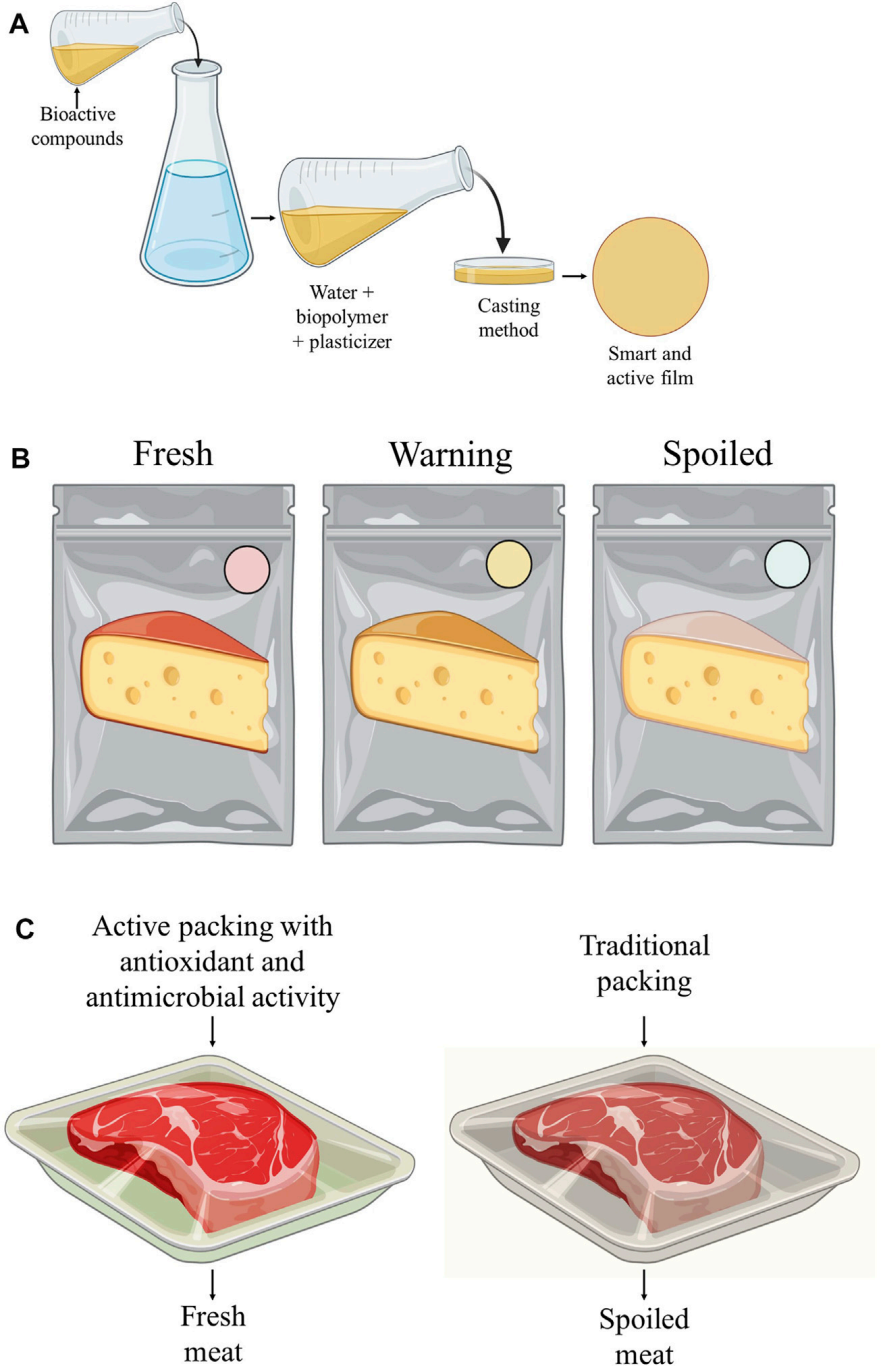
**(A)** production of films by casting. **(B)** application as smart packaging, and **(C)** application as active food packaging.

Other components, such as betalain anthocyanins, curcumins, tannins, and carotenoids, commonly applied in foods as natural colorings (Luzardo-Ocampo et al., 2021), have also been used for the development of active and smart biodegradable food packaging (Figure 4) (Alizadeh-Sani et al., 2020). Anthocyanins extracted from the residue of processing blueberry juice, for example, have already been used in the production of smart films using cassava starch capable of monitoring the quality of orange juice, corn oil, and chicken pieces. Anthocyanin acts as an indicator of pH change during storage, as its color is altered due to structural changes when there is pH variation (Luchese et al., 2018).

Anthocyanin extracted from black plum bark is also efficiently applied in films based on chitosan and TiO_2_, where incorporation results in high barrier properties against water vapor and UV-vis light and better mechanical strength (Zhang et al., 2019). In addition, it results in a higher free radical scavenging capacity and antimicrobial activity (*Escherichia coli*, *Staphylococcus aureus, Salmonella*, and *Listeria monocytogenes*), besides promoting the production of films capable of eliminating ethylene with potential application in pH-sensitive foods by detecting their changes and causing a color change.

Betacyanins extracted from the shell of dragon fruits can also be used to monitor the quality of fish freshness through their incorporation into intelligent packaging based on glucomannan–polyvinyl alcohol (Ardiyansyah et al., 2018). The presence of betacyanins also promotes a noticeable change from purple to yellow coloration due to the deterioration process of the product, which is, consequently, accompanied by increased levels of total volatile basic nitrogen (TVBN).

Protein hydrolysates, obtained through the enzymatic hydrolysis of proteins from agro-industrial by-products, have also been used as active agents in food films. Active food films based on alginate and protein hydrolysates obtained from the by-product of cottonseed oil extraction promote the formation of films with excellent visible light barrier properties, antioxidant activity, and antimicrobial potential against *S. aureus*, *Colletotrichum gloeosporioides,* and *Rhizopus oligosporus* (Oliveira-Filho et al., 2019).

Another prominent use of by-products is in the production of nutraceuticals, which are bioactive compounds used to meet the body’s needs and usually consumed in pharmaceutical preparations, such as pills, tablets, capsules, powders, and bottles (Kumar et al., 2017). Among the most commonly marketed and used nutraceuticals are amino acids, carotenoids (β-carotene, lutein, zeaxanthin, and lycopene), fatty acids (omega-3 and omega-6), minerals (copper, selenium, and zinc), polyphenols, vitamins (C and E), and several others (Souyoul et al., 2018), which can be extracted from agro-industrial by-products.

The possibility of applying bioactive components in food products and in new technologies to promote food quality and safety is huge. Due to the diversity of compounds and their possible interactions and diverse activities, each component must be properly evaluated to produce food, beverages, and active and smart packaging applied to food to guarantee maximum potential in the applications.

## 7 Conclusion and Future Directions

Large amounts of agro-industrial residues are generated during the processing of animal and vegetable materials, making it extremely necessary to adopt strategies for the integral use of residues or, even, for conversion into higher-value-added products. Biological approaches have several advantages compared to nonbiological processes, including the provision of extracts with high quality and high bioactivity, as well as with low toxicity.

Bioactive compounds obtained from by-products using the biological approach can be applied to develop foods and active or smart agents for biodegradable materials and packaging while contributing to consumer health, food safety, and sustainable use of natural resources.

That is why the biological approach is an important tool that must be continually improved and encouraged, especially due to the diversity of components that can be produced and the possible interactions and varied activities; each component must be properly evaluated to guarantee maximum potential in the applications.
